# Special Issue “Targeting Oxidative Stress for Disease: 2nd Edition”

**DOI:** 10.3390/ijms262010126

**Published:** 2025-10-17

**Authors:** Nada Oršolić, Maja Jazvinšćak Jembrek

**Affiliations:** 1 Division of Animal Physiology, Faculty of Science, University of Zagreb, Rooseveltov trg 6, HR-10000 Zagreb, Croatia; 2 Laboratory for Protein Dynamics, Division of Molecular Medicine, Ruđer Bošković Institute, Bijenička cesta 54, HR-10000 Zagreb, Croatia; maja.jazvinscak.jembrek@irb.hr; 3 School of Medicine, Catholic University of Croatia, Ilica 244, HR-10000 Zagreb, Croatia

Oxidative stress (OS) arises from a redox imbalance characterized by elevated levels of reactive oxygen species (ROS). It plays a crucial role in the pathogenesis of a wide range of seemingly unrelated conditions, including type 2 diabetes, cancer, neurological disorders, aging, cardiovascular diseases (such as hypertension, atherosclerosis, coronary artery disease, and heart failure), acute renal failure, preeclampsia, asthma, chronic obstructive pulmonary disease, rheumatoid arthritis, glaucoma, osteoporosis, and sexual dysfunction, among other things [1,2,3,4].

Building on the success of the first edition, this Special Issue continues to provide insights into the role of ROS-driven mechanisms in physiological processes and therapeutic strategies.

OS plays a central role in the onset and progression of neurodegenerative diseases [5], for which treatment options remain largely symptomatic and lack effective disease-modifying effects [6]. Because these disorders are driven by complex and interconnected cellular and molecular mechanisms, it is generally assumed that future therapeutic approaches must target several pathways simultaneously to be effective [7]. In that regard, various phytochemicals, especially those with polyphenolic structure, are promising candidates due to their multimodal activities. These include antioxidative, anti-inflammatory, anti-aggregation and mitochondria-protective effects, together with the capacity to restore communication between intracellular signaling pathways, particularly those that are redox sensitive [8,9,10]. However, challenges remain, particularly in optimizing brain delivery. Advances in nanotechnology hold promise for enhancing the bioavailability, targeted delivery, and controlled release of natural compounds, potentially maximizing therapeutic efficacy while minimizing long-term side effects [11].

Phytotherapy also shows potential in treating cardiomyopathies of various etiologies [12,13]. Polyherbal formulations, in particular, may be effective due to the synergistic interactions of their bioactive plant components, which together reduce oxidative damage and inflammation [14].

The benefits of natural compounds extend to women’s health as well. Their inclusion in intimate care products could help restore vaginal microbiota and epithelium and eliminate pathogenic microorganisms based on strong antioxidant, inflammatory, and anti- irritation properties of phytochemicals [15].

Epidemiological and toxicological analyses consistently demonstrate that the harmful effects of smoking are linked to oxidative damage [16]. Pregnancy itself is related to increased metabolic and oxygen demands, and maternal smoking further heightens fetal oxidative/nitrosative stress. Toxic smoke components are particularly detrimental to circulating red blood cells, impairing their function and membrane integrity [17,18]. Fungal infections, acting as additional stressors, may exacerbate these harmful effects on maternal and fetal erythrocytes by altering their redox status through secreted fungal proteins [19].

Exposure to cigarette smoke also promotes the development of lung cancer. Beyond environmental factors, oxidative stress, transcription factors, non-coding RNAs, and dysregulated cell signaling pathways all contribute to tumor initiation, therapy resistance and metastatic progression. A deeper understanding of the complex interplay between redox mechanisms, transcriptional regulation and non-coding RNAs may open new avenues for personalized cancer therapies [20]. Of particular interest are non-coding RNAs, such as antisense oligonucleotides and small interfering RNAs, which are being investigated as potential targets against cancer. Nevertheless, significant challenges remain, particularly related to delivery, specificity and tolerability [21].

The interaction between oxidative stress and non-coding RNAs is also relevant in osteoarthritis, where impaired mitochondrial function and enhanced levels of ROS have been observed in osteoarthritis chondrocytes. Non-coding RNAs have been considered as potential targets for novel therapeutic approaches, as they regulate key signaling pathways related to antioxidative defense (e.g., Nrf2/HO-1) and inflammation (e.g., NF-κB, MAPK, Wnt/β-catenin, TGFβ/Smad) [22,23]. Natural compounds have demonstrated protective effects in this context, particularly by regulating Nrf2/ARE signaling [24].

Despite growing knowledge of the molecular mechanisms underlying oxidative stress-related diseases, including neurodegenerative ones, therapeutic options are still limited and face numerous challenges. In the brain, for instance, the blood–brain barrier represents a major obstacle to stable drug delivery. Advances in nanotechnology, especially the development of nanoenzymes, artificial nanomaterials with enzyme-like ROS-scavenging properties, are promising for overcoming these barriers. Their application in treating neurological diseases is increasing [25], and further research may yield more efficient nanoenzymes and other nanomaterials capable of restoring redox homeostasis, attenuating disease progression, and ultimately improving human health across a variety of conditions.
